# Epidemiology of dialysis-treated end-stage renal disease patients in Kazakhstan: data from nationwide large-scale registry 2014–2018

**DOI:** 10.1186/s12882-020-02047-6

**Published:** 2020-09-21

**Authors:** Abduzhappar Gaipov, Alpamys Issanov, Kainar Kadyrzhanuly, Dinara Galiyeva, Marina Khvan, Mohamad Aljofan, Miklos Z. Molnar, Csaba P. Kovesdy

**Affiliations:** 1 Department of Clinical Sciences, Nazarbayev University School of Medicine, Kerey and Zhanibek Khans Street 5/1, Room 345, Nur-Sultan City, Kazakhstan; 2 Department of Biomedical Sciences, Nazarbayev University School of Medicine, Nur-Sultan, Kazakhstan; 3 Division of Nephrology, Department of Medicine, University of Tennessee Health Science Center, Memphis, TN USA; 4 James D. Eason Transplant Institute, Methodist University Hospital, Memphis, TN USA

**Keywords:** Dialysis, Epidemiology, ESRD, Kazakhstan, Registry

## Abstract

**Background:**

The epidemiology of dialysis patients has been little studied in developing countries and economies in transition. We examined the prevalence, incidence and mortality rate of dialysis patients in Kazakhstan, via aggregation and utilization of large-scale administrative healthcare data.

**Methods:**

The registry data of 8898 patients receiving dialysis therapy between 2014 and 2018 years were extracted from the Unified National Electronic Health System (UNEHS) and linked with the national population registry of Kazakhstan. We provide descriptive statistics of demographic, comorbidity and dialysis-related characteristics.

**Results:**

Among all patients undergoing maintenance dialysis for end-stage renal disease (ESRD), there were 3941 (44%) females and 4957 (56%) males. 98.7% of patients received hemodialysis and 1.3% peritoneal dialysis. The majority of the patients (63%) were ethnic Kazakhs, 18% were Russians and 19% were of other ethnicities. The prevalence and incidence rate in 2014 were 135.2 and 68.9 per million population (PMP), respectively, which were different in 2018 [350.2 and 94.9 PMP, respectively]. Overall mortality rate among dialysis patients reduced from 1667/1000 patient-years [95%Confidence Interval (CI): 1473–1886] (PY) in 2014 to 710/1000PY [95%CI: 658–767] in 2018. We observed 13% lower crude survival probability in females compared to males and in older patients compared to younger ones. Russian ethnicity had 58% higher risk of death, while other ethnicities had 34% higher risk of death compared to in those of Kazakh ethnicity.

**Conclusion:**

We describe for the first time in Kazakhstan an increase in the prevalence and incidence of ESRD on dialysis, while mortality rate decreased over time, during 2014–2018. We observed statistically significant lower survival probability in female dialysis patients compared to males, in older patients compared to younger ones, and in patients of Russian ethnicity compared to Kazakh.

## Background

Kazakhstan is a central Asian country with a fast-growing economy and a multiethnic population of 18.6 million. The major ethnicities in Kazakhstan are ethnic Kazakh, who make up the majority of the population with 67.5% followed by ethnic Russians 19.5% and the remainder of the population made up of several other ethnicities including Uzbeks, Uyghurs, Ukrainians, and Koreans [1]. The World Health Organization (WHO) estimates the average life expectancy in Kazakhstan to be 64 years (59 years for males and 70 years for females) [2]. According to the WHO health profile for Kazakhstan, non-communicable chronic diseases (cardiovascular diseases, cancer, chronic respiratory disease and diabetes) accounted for 86% of all deaths [2]. The prevalence of diabetes was reported as 12% and the prevalence of hypertension was estimated to be 27% in the general population [3]. Interestingly, several reports about the prevalence of non-communicable diseases in Kazakhstan, including that of the WHO reported a rapid increase in the burden of chronic kidney disease (CKD) [2, 4, 5].

Globally, the estimated number of individuals affected by kidney disease exceeds 850 million with 843.6 million accounted for by CKD [6]. Recent data showed that globally the incidence and prevalence of CKD during years 1990–2016 increased by 89 and 87%, respectively, surpassing 100% in countries with middle and low sociodemographic index (SDI) [5]. There was an estimated two-fold increase in the number of deaths from CKD over the last 3 decades, which shifted CKD from being the 18th top cause of death in the year 1990 to the 11th in the year 2016 [4, 5, 7].

The Global Burden of Disease Study, which is the only source of data for Kazakhstan, reported the number of CKD patients to be as high as one million or 5.5% of the general population in 2016, which correlates with a 44% increase in the number of CKD cases between 1990 and 2016, including 1600 deaths, equaling approximately 1% of the total deaths that occurred in 2016 [2, 5].

There is a lack of epidemiological information of end-stage renal disease (ESRD) in Kazakhstan. Despite the availability of chronic hemodialysis treatment since 1974, the number of public dialysis centers were limited to the big cities, but legislative changes in the early 2000s allowed the establishment of private dialysis centers throughout the country, which resulted in improved patient access to such facilities. The establishment of the Unified National Electronic Health System (UNEHS) in 2014, made epidemiological data of dialysis patients readily available for healthcare researchers and providers [8, 9].

Therefore, the objective of the current study is to use the large-scale administrative health data available in the UNEHS to estimate the prevalence, incidence and mortality rates of end-stage renal disease patients receiving dialysis in Kazakhstan. Data from a five-year period, inclusive from the date of establishment of UNEHS, 2014 until the latest available, which is 2018 was collected and statistically analyzed to determine each of the parameters.

## Methods

### Study population

The study population consisted of ESRD patients included in the database, “Registry of Chronic Kidney Disease” (RCKD) within UNEHS that was established in 2014. The raw data of 11,087 patients receiving renal replacement therapy (hemodialysis, peritoneal dialysis and kidney transplantation) between 2014 and 2018 were extracted from RCKD as Microsoft Excel file. Initially, 310 duplicate patient records were removed if population registry number (RPN ID) were similar. Depending on primary disease ICD-10 codes, patients who underwent dialysis were labeled as either AKI (ICD-10 code: N17) or ESRD (ICD-10 code: N18). Patients with AKI (*n* = 1421) and those patients who underwent preemptive kidney transplantation (*n* = 458) were dropped from the cohort and 8898 chronic dialysis patients were included for further statistical evaluation. 1437 out of 8898 patients had started dialysis treatment before January 1, 2014, and included to the current cohort. The detailed information about *data sources* described in supplementary material. The number of population growth in whole Kazakhstan and its regions were obtained from the source of Statistics committee under the Ministry of National Economy of the Republic of Kazakhstan [1].

### Exposures and covariates

Individual patient data included date of birth, gender, ethnicity, and address by region, ICD-10 diagnosis, date of first dialysis and type of dialysis, and date of kidney transplantation. The date of the first dialysis procedure and the date of kidney transplant surgery might be registered to the UNHS retrospectively from previous temporary electronic systems and old registries if they were conducted before 2014. Information about date of birth and death (if any) were obtained through linkage with the Population Registry through population registry number (RPN number). Age was categorized in five groups (below 18 years old (y.o.), 18–34 y.o., 35–50 y.o., 51–70 y.o. and above 70 y.o.), ethnicity to Kazakhs, Russians and others (included Uzbeks, Uyghurs, Ukrainians, Koreans, and other 37 ethnicities); and education level was categorized into five groups according to International Standard Classification of Education 11 (ISCED) as ISCED-2 (Lower Secondary Education), ISCED-3 (Upper Secondary Education), ISCED-4 (Post-secondary non-Tertiary Education) and ISCED-5 (Short-cycle tertiary education) [10].

### Outcome assessment

The prevalence, incidence and all-cause mortality of dialysis patients were assessed. Prevalence of ESRD patients were studied for five consecutive years (2014–2018). For each year, a period prevalence was calculated by dividing all alive patients receiving dialysis at any point of the year by the average total general population size during the year Similarly, incidence and mortality were calculated by dividing the number of new patients and deaths, respectively, by the average total general population size for each year. The number of population growth in Kazakhstan overall and its regions were obtained from the Statistics Committee [1]. All-cause mortality data, censoring events such as kidney transplantation, and associated dates were obtained from RCKD and the population registry. The start of the follow-up period was the date of dialysis therapy initiation, and patients were followed up until death or other censoring events, including kidney transplantation or end of the follow-up period (December 31st, 2018).

### Statistical analysis

Data are summarized as percentages for categorical variables. Kaplan-Meier estimation and log-rank test were used to calculate crude survival and statistically significant differences in survival by age groups, gender, ethnicity and education. Cox proportional hazards regression analysis, after checking its assumptions, was used to obtain crude and adjusted hazard ratios. Cumulative incidence curves and competing risk regression analysis by Fine and Gray [11] were used to assess the transplant censored all-cause mortality between age groups, gender, ethnicity and education level.

Mortality rate within the dialysis population is presented as per 1000 patient year (/1000PY) in different groups. Statistical analysis, data cleaning (identifying and removing duplicate cases) and data management (labeling all data, creating and categorizing new variables) was performed using STATA 15 MP/IC Version (STATA Corporation, College Station, TX). *P* values are two-sided and reported as statistically significant at < 0.05 for all analyses. The study was approved by the Institutional Review Ethics Committee (NU-IREC 203/29112019), with exemption from informed consent.

## Results

### Demographic data

The demographic information of the cohort are provided in Table 1. During 2014–2018, there were a total of 3941 (44%) female and 4957 (56%) male patients who underwent maintenance dialysis (98.7% hemodialysis and 1.3% peritoneal dialysis). 63% of patients were ethnic Kazakhs, 18% were of Russian ethnicity and 19% were listed as other ethnicities. 7.7% (684) of patients underwent kidney transplantation after initiation of maintenance dialysis. During a median 1.88-year (684 (IQR: 228–1362) days) follow-up period after dialysis initiation there were 2692 deaths (29%) in the overall cohort.

**Table 1:** Demographic data and crude mortality rate per 1000 patient years

Variables	*N* = 8898 (%)	Mortality rate per 1000 patient year [95%CI]
Gender		
Female	3941 (44.3%)	137 [130–145]
Male	4957 (55.7%)	118 [112–125]
Age, groups		
< 18 y.o.	100 (1.1%)	16 [7–36]
18–34 y.o.	1460 (16.4%)	48 [42–55]
35–50 y.o.	2308 (25.9%)	85 [78–93]
51–70 y.o.	4279 (48.1%)	173 [165–182]
> 70 y.o.	751 (8.4%)	343 [311–379]
Ethnicity		
Kazakh	5574 (62.8%)	108 [102–113]
Russian	1570 (17.7%)	173 [160–188]
Others	1726 (19.5%)	150 [138–163]
Education		
ISCED-2	224 (2.5%)	165 [132–206]
ISCED-3	1673 (18.8%)	137 [125–149]
ISCED-4	1805 (20.3%)	132 [121–143]
ISCED-5	843 (9.5%)	97 [85–111]
Missing	4353 (48.9%)	NA
Dialysis modality		
Peritoneal dialysis	112 (1.3%)	133 [92–191]
Hemodialysis	8657 (98.7%)	129 [124–134]
Kidney transplant		
No	8214 (92.3%)	144 [139–150]
Yes	684 (7.7%)	12 [9–17]
Outcome		
Follow-up period, days	684 (228–1362)	NA
Alive	6206 (69.7%)	NA
Died	2692 (30.3%)	NA

*Abbreviations*: *ISCED* International Standard Classification of Education

### Prevalence, incidence and mortality per million population

**Fig. 1:**
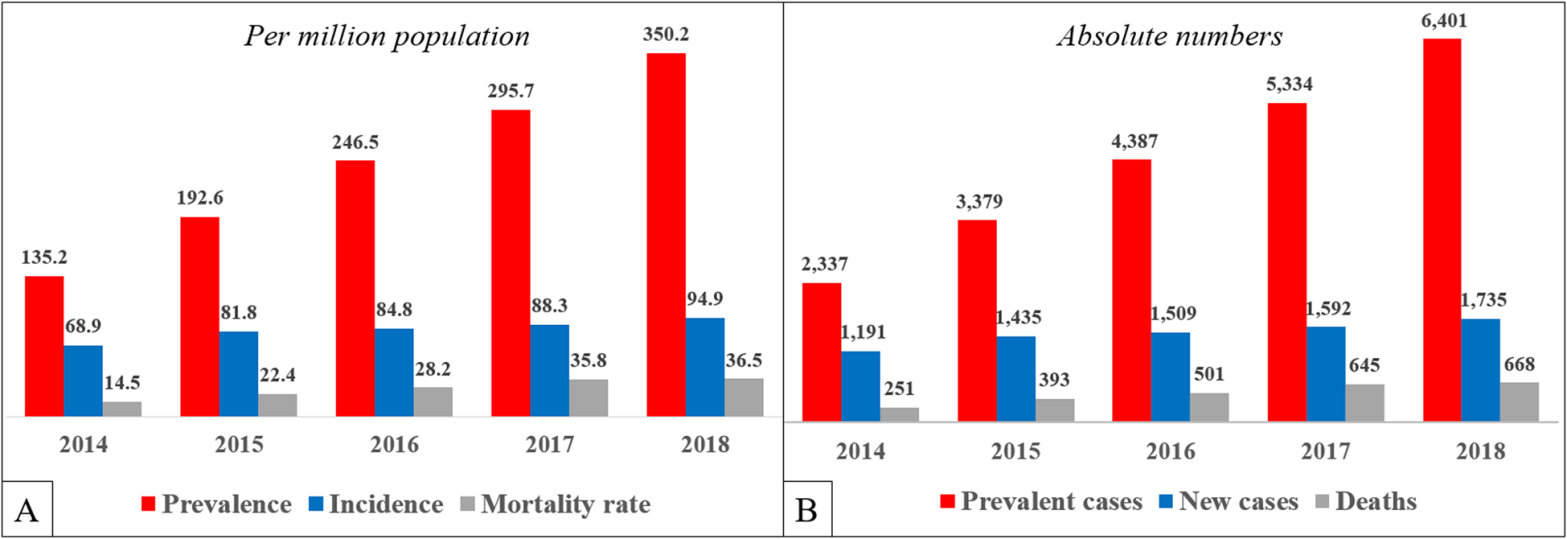
Prevalence, incidence and mortality of dialysis patients for 2014–2018 years

The prevalence and incidence (Fig. 1a) in 2014 were 135.2 and 68.9 per million population (PMP) respectively and differed from 2018 (350.2 and 94.9 PMP respectively). The number of prevalent patients dramatically increased from 2337 to 6401 during 2014–2018 (Fig. 1b) in both urban and rural population (Supplement Figure 1), especially in patients within the 51–70 y.o. age category (Supplement Figure 2), despite a small population growth of one million over the same time period (from 17.2 to 18.2 million) (Supplement Table 1). Due to the increase in the prevalence of ESRD in the general population, the number of deaths attributable to ESRD also increased in the population as a whole during 2014–2018 (14.5 vs. 36.5 deaths PMP, Fig. 1a). Among all the regions of Kazakhstan, the South and North Kazakhstan regions had the highest mortality (> 40 PMP) rate in 2017 (Supplement Figure 3).

### Mortality rate among dialysis population

**Fig. 2:**
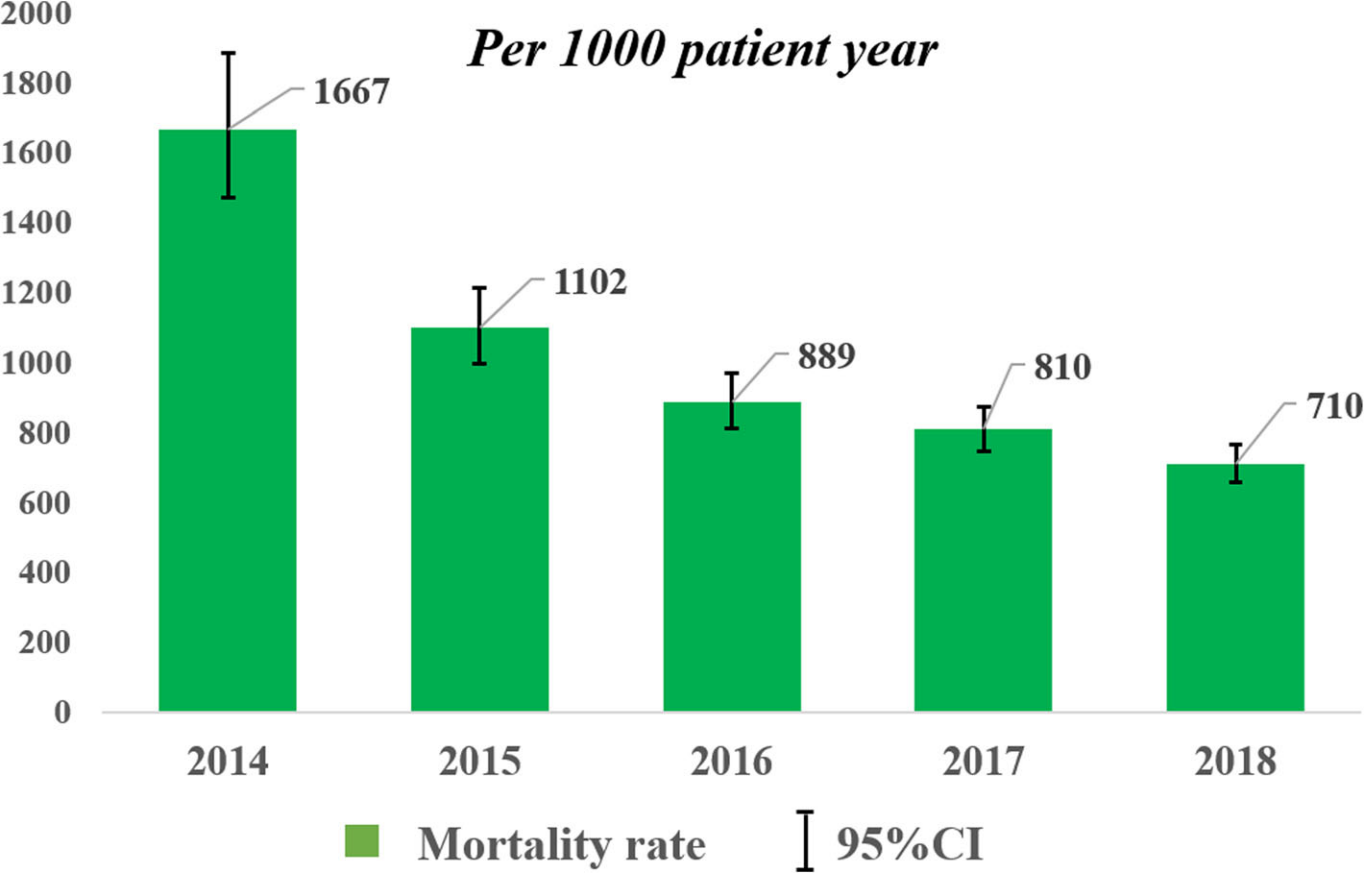
Crude mortality rate among dialysis population for 2014–2018 years

The crude mortality rate per thousand patient year (/1000PY) is provided in Table 1. The crude mortality rate was higher in females compared to males (137/1000PY [95%CI: 130–145] vs 118/1000PY [95%CI: 112–125]), in those of Russian ethnicity compared to Kazakhs (173/1000PY [95%CI: 160–188] vs 108/1000PY [95%CI: 102–113]) and in older patients compared to younger ones (Table 1). Despite increased mortality from ESRD in whole population (Fig. 1a), the mortality rate among dialysis patients ultimately decreased from 1667/1000PY [95%CI: 1473–1886] in 2014 to 710/1000PY [95%CI: 658–767] in 2018 (Fig. 2).

### Survival probability overall and by gender, ethnicity, age and educational level

**Fig. 3:**
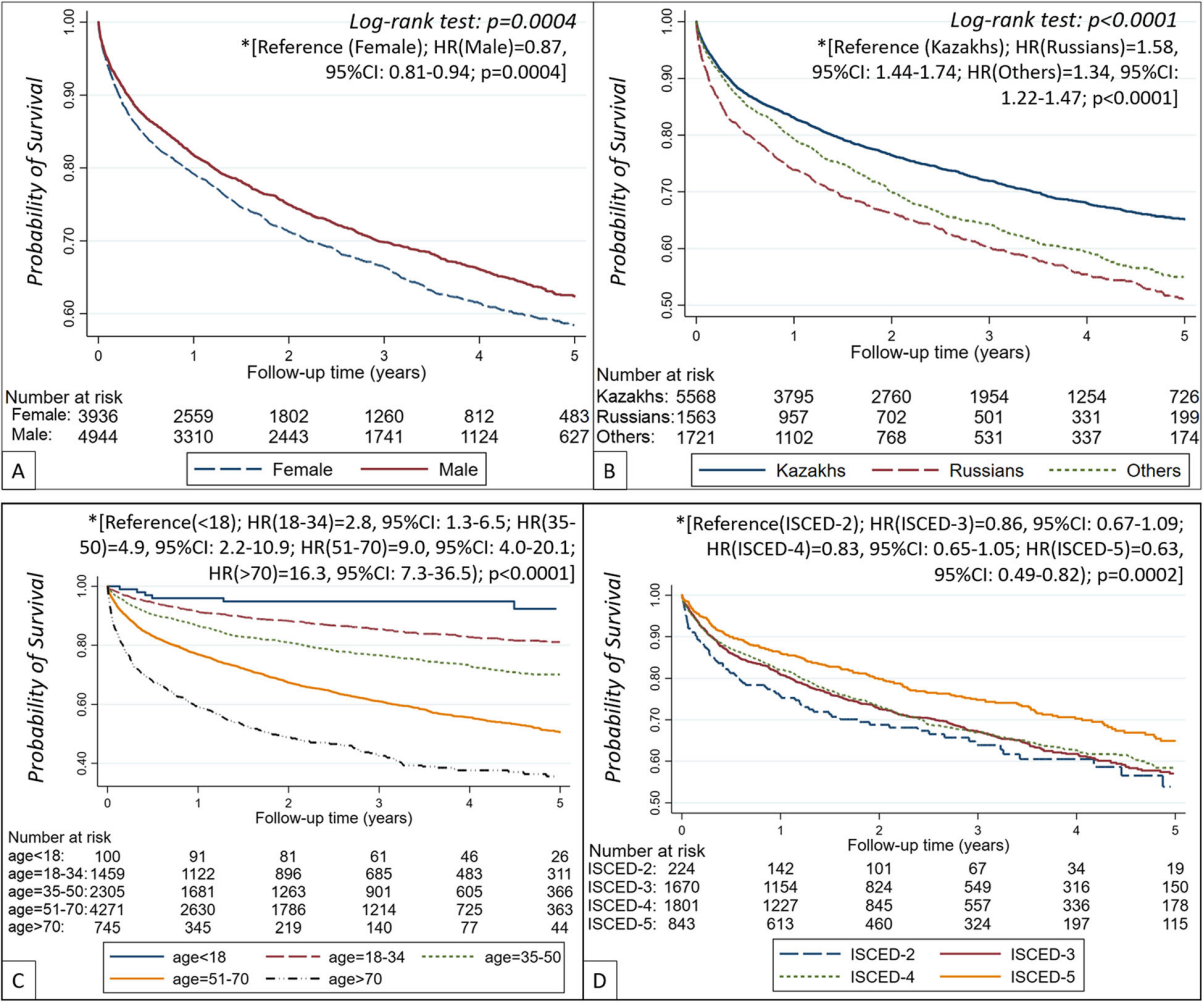
Crude survival probability on dialysis by gender (**a**), ethnicity (**b**), age category (**c**) and education level (**d**). *Unadjusted Cox proportional hazard regression analysis. Abbreviations: ISCED - International Standard Classification of Education; HR – hazard ratio

In Kaplan-Meier analyses (Fig. 3a) we observed a 13% (HR = 0.87 [95%CI: 0.81–0.94], *p = 0.0004*]) lower crude survival probability in females compared to males, which remained similar after adjustment for age (Supplement Figure 4A). Patients of Russian ethnicity had 58% higher risk of death, while other ethnicities had 34% higher risk of death compared to in those of Kazakh ethnicity (Fig. 3b). Even after adjustment for age and gender (Supplement Figure 4B), Kazakhs (reference) had statistically significant higher survival probability than Russian and other ethnicities (HR_(Russian)_ = 1.29 [95%CI: 1.17–1.42]; HR_(Others)_ = 1.16 [95%CI: 1.05–1.28]; *p < 0.0001,* respectively).

Compared to patients < 18 y.o., patient in the 18–34, 35–50, 51–70 and > 70 y.o. groups had 2.8-fold, 4.9-fold, 9.0-fold and 16.3-fold higher risk of death, respectively (Fig. 3c). Also, patients with ISCED-2 educational level (reference) had lower survival probability (Fig. 3d) compared to those with ISCED-3, ISCED-4 and ISCED-5 education levels (HR = 0.86 [95%CI: 0.67–1.09]; HR = 0.83 [95%CI: 0.65–1.05] and HR = 0.63 [95%CI: 0.49–0.82]*,* respectively).

### Transplant censored all-cause death by gender, ethnicity, age and educational level

**Fig. 4:**
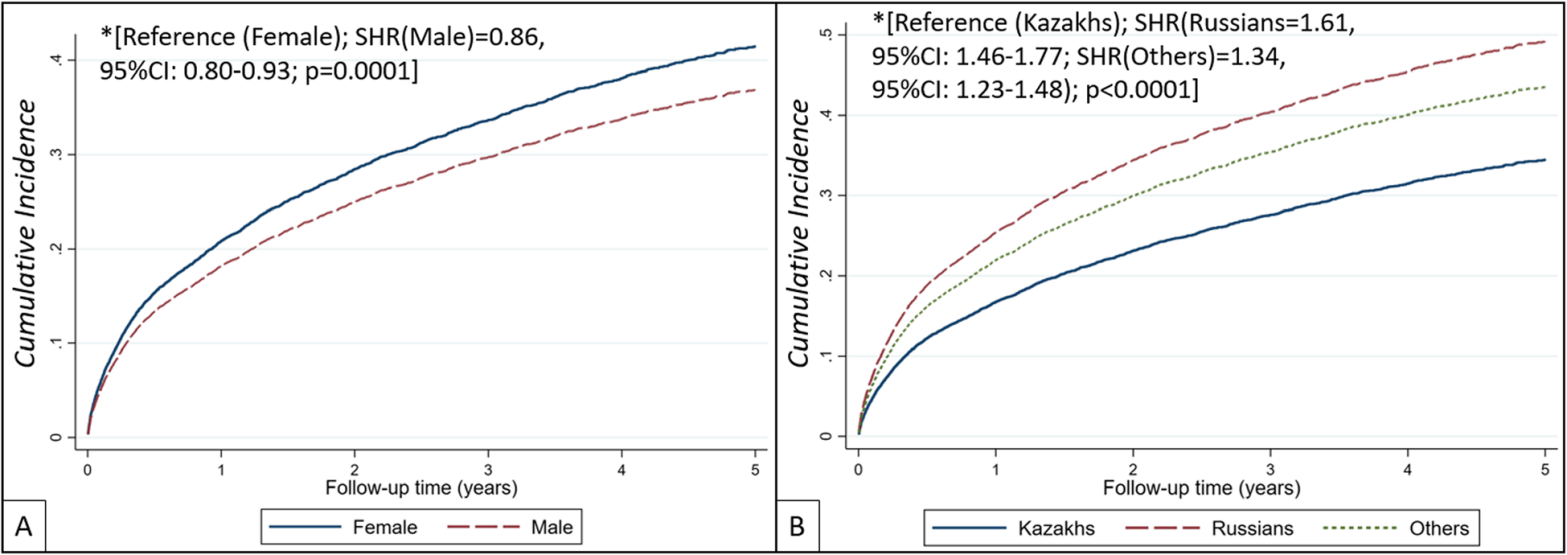
Cumulative incidence of transplant censored all-cause death on dialysis by gender (**a**) and ethnicity (**b**). *Unadjusted competing risk regression analysis. Abbreviations: SHR – subdistribution hazard ratio

A similar trend was observed for transplant censored all-cause deaths. The cumulative incidence of death was higher in females, compared to males, whereas the cumulative incidence of death was substantially lower in patients of Kazakh ethnicity, compared to Russians and other ethnicities (Fig. 4). In unadjusted competing risk regression analysis, compared to females (references) male gender was associated with 14% lower risk of death (SubHazard Ratio (SHR) = 0.86 [95%CI: 0.80–0.93]; *p* < 0.0001), as well as compared to Kazakh ethnicity (reference) Russian and Other ethnicities had 61 and 34% higher chance of death (SHR_(Russian)_ = 1.61 [95%CI: 1.46–1.77]; SHR_(Others)_ = 1.34 [95%CI: 1.23–1.48]; *p < 0.0001,* respectively). Results remained statistically significant after adjustments (Supplement Figure 5). Finally, the cumulative incidence of death on dialysis by age category (Supplement Figure 6A) and education level (Supplement Figure 6B) were similarly to survival probabilities calculated by the Kaplan-Meier method. Competing risk regression analysis showed that older patients had statistically significant higher risk of death compared to young ones and patients with higher educational levels (ISCED-3, ISCED-4 and ISCED-5) had reduced risk death compared to those with the lowest educational level (ISCED-2).

## Discussion

This is the first central Asian study to investigate the epidemiology of dialysis patients using large-scale administrative health data that has recently become available in Kazakhstan. The results showed an increase in the prevalence and incidence rates and decrease in mortality rate of dialysis patients over the last 5 years. The survival probability was statistically significant lower for females compared to males. Interestingly, after adjustment for age and gender, a statistically significant lower survival probability of ethnic Russians or other compared to Kazakh ethnicities was shown.

The current findings are in line with information recently published USRDS Annual reports that also described the epidemiology of ESRD in Kazakhstan (in international comparison section) that was conducted by call-based data collection from all dialysis providers [12–14]. However, we were not able to access demographic and mortality data during this call-based data collection.

The expenses related to all modalities of renal replacement therapy (hemodialysis, peritoneal dialysis and kidney transplantation) are fully covered by government for citizens of Kazakhstan from the beginning [14, 15]. There was no therapy available in Kazakhstan for renal anemia, mineral-bone diseases, and other ESRD related complications during the 1990s due to lack of medications and their registration in the country. However, the implementation of public-private partnership in healthcare during the early 2010s substantially improved patient access to dialysis in wider geographic regions including rural areas [16, 17]. This may explain the increasing incidence of dialysis patients beginning from 2013. To date, the incidence and prevalence PMP is reported to be higher around big cities and wealthier parts of the country. This could be due to the fact that these areas might have better access to dialysis services than other regions such as rural or isolated and underdeveloped villages. However, limited access to dialysis in rural areas in earlier years may have resulted in the accumulation of a large number of untreated or undiagnosed patients, thus these patients were likely included in the dialysis centers upon their later establishment. Our findings demonstrated that the prevalence and incidence has continuously increased in both rural and urban regions. On the other hand, several private ambulatory dialysis centers started to emerge in all rural regions of Kazakhstan during the last couple of years. Consequently, the availability of these centers and the inclusion of previously undiagnosed patients have likely contributed to the increased incidence rate.

Another factor affecting the described secular changes is the implementation of state healthcare programs for the management of cardiovascular diseases in 2005, which aimed to reduce cardiovascular mortality by developing heart and stroke centers [18, 19]. This may have led to an increase in the life expectancy of patients with CKD, who would then survive to ESRD. The development of national guidelines on CKD management and dialysis treatment based on international guidelines that begun in the early 2010s substantially improved dialysis adequacy and decreased ESRD-related complications, which could have resulted in an increase in the survival of dialysis patients, and thus may have impacted on the overall prevalence rate in the following years. Our findings demonstrate that while the prevalence and incidence of ESRD were continuously increasing, there was a substantial reduction in the mortality rate of dialysis patients during the same period. These trends indicate an overall beneficial impact of the aforementioned healthcare reforms, affording better access to dialysis while improving the health outcomes among the citizens of Kazakhstan with CKD and ESRD.

While there was no difference in the prevalence or incidence rate between males and females, the crude probability of survival for females was much lower than their male counterparts. Studies examining the association between patient gender and survival rate have shown conflicting results. For instance, Artan et al., who reported the results of an observational study that investigated whether or not there is an association between patient gender and mortality rate among dialysis patients, claimed that there is no difference in survival rates between male and female hemodialysis patients [20]. However, different studies including that of Bloembergen et al., which investigated the causes of death in dialysis patients between the different sex, reported that males had a 22% higher risk of death than females, which the authors attributed to concomitant diseases such as cardiovascular disease [21]. Consequently, the current study is not the first study to report a difference in survival rate between male and female dialysis patients, but it is the first to show that females have a lower survival rate than males. Possible reasons might be later referral to dialysis, more severe burden of ESRD complications (renal anemia, cardiovascular disease, etc.), or country-specific sociodemographic factors. However, we cannot infer causality from the current database, and hence these observations require further focused examination.

Also, the results showed that ethnic Russians experience higher mortality rate on dialysis than other ethnicities. While we did not investigate the reasons for the observed difference, the results seem to be in agreement with previous studies that reported higher mortality rate among ethnic Russians in central Asia compared to other ethnicities, including Davletov and colleagues who studied mortality rates in Kazakhstan by ethnic group and the most commonly reported contributing factors [22]. The study reported that despite having higher levels of education and socioeconomic status than Kazakhs, ethnic Russians in Kazakhstan appear to have a higher mortality rate than other ethnicities. Destructive lifestyle such as alcohol consumption, heavy smoking, high-fat diet and lack of physical activity, are claimed to be the major contributing factors for the higher mortality rate amongst Russians [23]. However, the important question, whether there are other contributing factors besides lifestyle warrants further investigation.

There are several limitations in the current study such as the lack of cause-specific mortality data, possible errors with disease coding, and potential loss of follow-up for patients who transitioned between kidney transplants and dialysis by leaving the country. Also, residual confounding is likely present in the study results as the registry data was limited to few variables which could be adjusted for. However, this is the first study from the Central Asia and Eurasian regions to demonstrate the epidemiology of dialysis patients based on a compiled nationwide digital healthcare data from Kazakhstan. The findings warrant further investigations into patient mortality rate based on age, gender and ethnicity in Kazakhstan and the region.

## Conclusion

This the first study in Kazakhstan and possibly in the entire central Asia region, to examine the prevalence, incidence and mortality rate of dialysis patients. The current study analyzed large-scale administrative healthcare care data of dialysis patients over a five-year period between 2014 and 2018. The results showed an increase in the prevalence and incidence of dialysis patients, who accounted for a rising proportion of deaths within the general population. Meanwhile, the mortality rate within the dialysis population displayed a marked decrease of 43%, suggesting improvement in access to care and/or better healthcare practices. We described a lower survival rate for females compared to males, of older patients compared to young ones and patients of Russian ethnicity compared to other ethnicities.

## Supplementary information

**Additional file 1: Data resources**. **Table S1.** Average population of Kazakhstan and by region between 2014 and 2018 years. **Figure S1.** Prevalence, incidence and mortality of dialysis patients by rural and urban regions for 2014–2018 years. **Figure S2.** The number of new cases by age category for the period 2014–2018. **Figure S3.** Prevalence (A), Incidence (B) and Mortality (C) of Dialysis patients by Regions in 2017. **Figure S4.** Adjusted survival probability on dialysis by gender (panel A, adjusted for age) and ethnicity (panel B, adjusted for age and gender). *Adjusted Cox proportional hazard regression analysis. Abbreviations: HR – hazard ratio. **Figure S5.** Adjusted cumulative incidence of transplant censored all-cause death on dialysis by gender (panel A, adjusted for age) and ethnicity (panel B, adjusted for age and gender). *Adjusted competing risk regression analysis. Abbreviations: SHR – subdistribution hazard ratio. **Figure S6.** Cumulative incidence of transplant censored all-cause death on dialysis by age category (A) and education level (B). *Unadjusted competing risk regression analysis. Abbreviations: ISCED - International Standard Classification of Education; SHR – subdistribution hazard ratio.

## Data Availability

The data that support the findings of this study are available from Republican Center for Electronic Health of the Ministry of Health of the Republic of Kazakhstan, but restrictions apply to the availability of these data, which were used under the contract-agreement (#173–2020, from March 11th, 2020) for the current study, and so are not publicly available. Data are however available from the authors upon reasonable request and with permission of Ministry of Health of the Republic of Kazakhstan.

## Abbreviations

AKI: Acute kidney injury
CKD: Chronic kidney disease
CI: Confidence interval
ESRD: End-Stage Renal Disease
HR: Hazard ratio
ICD: International code of diseases
ISCED: International Standard Classification of Education
PMP: Per million population
PY: Patient year
RCKD: Registry of Chronic Kidney Disease
SDI: Low sociodemographic index
SHR: SubHazard Ratio
UNEHS: Unified National Electronic Health System
USRDS: United States Renal Data System
WHO: World Health Organization

## References

[1] Statistics committee Ministry of National Economy of the Republic of Kazakhstan (2019). Main Socio-Economic Indicators.

[2] Organization WH (2018). Noncommunicable Diseases (NCD) country profiles 2018: Kazakhstan.

[3] Saeedi P, Petersohn I, Salpea P, Malanda B, Karuranga S, Unwin N, Colagiuri S, Guariguata L, Motala AA, Ogurtsova K (2019). Global and regional diabetes prevalence estimates for 2019 and projections for 2030 and 2045: Results from the International Diabetes Federation Diabetes Atlas. Diabetes Res Clin Pract.

[4] Moraga P, Collaborators GCoD (2017). Global, regional, and national age-sex specific mortality for 264 causes of death, 1980-2016: a systematic analysis for the global burden of disease study 2016. Lancet.

[5] Xie Y, Bowe B, Mokdad AH, Xian H, Yan Y, Li T, Maddukuri G, Tsai CY, Floyd T, Al-Aly Z (2018). Analysis of the global burden of disease study highlights the global, regional, and national trends of chronic kidney disease epidemiology from 1990 to 2016. Kidney Int.

[6] Jager KJ, Kovesdy C, Langham R, Rosenberg M, Jha V, Zoccali C. A single number for advocacy and communication—worldwide more than 850 million individuals have kidney diseases. Nephrol Dial Transplant. 2019;34(11):1803–5. 10.1093/ndt/gfz174 31566230

[7] Dicker D, Nguyen G, Abate D, Abate KH, Abay SM, Abbafati C, Abbasi N, Abbastabar H, Abd-Allah F, Abdela J (2018). Global, regional, and national age-sex-specific mortality and life expectancy, 1950–2017: a systematic analysis for the global burden of disease study 2017. Lancet.

[8] Arynova Z, Baiguzhinova L (2019). Development of electronic healthcare in Kazakhstan as a factor of improving the quality of medical services. Fundam Appl Res Pract Leading Sci Sch.

[9] Kalimoldayev M, Belginova S, Uvaliyeva I, Ismukhamedova A. IT Infrastructure of e-Health of the Republic of Kazakhstan. In: Shokin Y,Shaimardanov Z. (eds) Computational and Information Technologies in Science, Engineering and Education. CITech 2018. Commun Comput and Inf Sci. 2019;998:54-63. 10.1007/978-3-030-12203-4_6.

[10] Schneider SL (2013). The international standard classification of education 2011. Comp Soc Res.

[11] Fine JP, Gray RJ (1999). A proportional hazards model for the subdistribution of a competing risk. J Am Stat Assoc.

[12] Saran R, Robinson B, Abbott KC, Agodoa LYC, Bragg-Gresham J, Balkrishnan R, Bhave N, Dietrich X, Ding Z, Eggers PW (2019). US Renal Data System 2018 Annual Data Report: Epidemiology of Kidney Disease in the United States. Am J Kidney Dis.

[13] Saran R, Robinson B, Abbott KC, Agodoa LYC, Bhave N, Bragg-Gresham J, Balkrishnan R, Dietrich X, Eckard A, Eggers PW (2018). US renal data system 2017 annual data report: epidemiology of kidney disease in the United States. Am J Kidney Dis.

[14] Baigenzhin A, Doskaliyev Z, Tuganbekova S, Zharikov S, Altynova S, Gaipov A (2015). Organ Transplants in Kazakhstan. Exp Clin Transplant.

[15] Tchokhonelidze I, Zemchenkov A (2019). Current status, challenges, and the role of ISN in advancement of nephrology in the newly independent states and Russia region. Kidney Int.

[16] Abdymanapov SA, Toxanova AN, Galiyeva AH, Abildina AS, Aitkaliyeva AM (2016). Development of public-private Partnership in the Republic of Kazakhstan. Int Electron J Math Educ.

[17] Amagoh F (2011). New public management and health reform in Kazakhstan. Int J Public Adm.

[18] Nugmanova A, Pillai G, Nugmanova D, Kuter D (2008). Improving the management of hypertension in Kazakhstan: implications for improving clinical practice, patient behaviours and health outcomes. Global Public Health.

[19] Bekbossynov S, Medressova A, Murzagaliyev M, Salov R, Dzhetybayeva S, Andossova S, Bekbossynova M, Pya Y (2014). Surgical heart failure treatment program-the experience of Kazakhstan. Giornale italiano di cardiologia (2006).

[20] Artan AS, Kircelli F, Ok E, Yilmaz M, Asci G, Dogan C, Oto O, Gunestepe K, Basci A, Sever MS (2016). Dialyzing women and men: does it matter? An observational study. Clin Kidney J.

[21] Bloembergen WE, Port FK, Mauger EA, Wolfe RA (1994). Causes of death in Dialysis patients - racial and gender differences. J Am Soc Nephrol.

[22] Davletov K, McKee M, Berkinbayev S, Battakova Z, Zhussupov B, Amirov B, Junusbekova G, Rechel B (2016). Ethnic differences in all-cause mortality rates in Kazakhstan. Public Health.

[23] Davletov K, McKee M, Berkinbayev S, Battakova Z, Vujnovic M, Rechel B (2015). Regional differences in cardiovascular mortality in Kazakhstan: further evidence for the ‘Russian mortality paradox’?. Eur J Pub Health.

[24] Gaipov A, Issanov A, Kadyrzhanuly K, Galiyeva D, Khvan M, Molnar M, Kovesdy C (2020). SAT-208 epidemiology of dialysis patients in Kazakhstan: data from nationwide large-scale registry 2014-2018. Kidney Int Rep.

