# Supplementary material for: Epidemiology of dialysis-treated end-stage renal disease patients in Kazakhstan: data from nationwide large-scale registry 2014–2018
Source: BMC Nephrol. 2020 Sep 21;21:407. doi: 10.1186/s12882-020-02047-6 (PMC7504636; doi:10.1186/s12882-020-02047-6)
Supplement: Supplementary file 1 — Additional file 1: Data resources. Table S1. Average population of Kazakhstan and by region between 2014 and 2018 years. Figure S1. Prevalence, incidence and mortality of dialysis patients by rural and urban regions for 2014–2018 years. Figure S2. The number of new cases by age category for the period 2014–2018. Figure S3. Prevalence (A), Incidence (B) and Mortality (C) of Dialysis patients by Regions in 2017. Figure S4. Adjusted survival probability on dialysis by gender (panel A, adjusted for age) and ethnicity (panel B, adjusted for age and gender). *Adjusted Cox proportional hazard regression analysis. Abbreviations: HR – hazard ratio. Figure S5. Adjusted cumulative incidence of transplant censored all-cause death on dialysis by gender (panel A, adjusted for age) and ethnicity (panel B, adjusted for age and gender). *Adjusted competing risk regression analysis. Abbreviations: SHR – subdistribution hazard ratio. Figure S6. Cumulative incidence of transplant censored all-cause death on dialysis by age category (A) and education level (B). *Unadjusted competing risk regression analysis. Abbreviations: ISCED - International Standard Classification of Education; SHR – subdistribution hazard ratio. [file 12882_2020_2047_MOESM1_ESM.docx]

**SUPPLEMENTARY MATERIAL**

**Data resources**

The Republican Center of Electronic Healthcare (RCEH) is the governmental organization reported to Ministry of Healthcare. The main function of the RCEH is to promote the development and improvement of the digital electronic infrastructure of the healthcare system and medical statistics overall Kazakhstan. As a part of the electronic government, RCEH responsible to develop Unified National Electronic Health System (UNEHS). The UNEHS is the nationwide electronic health systems, including many information (digital) such as billing information, date of the provided health services, superficial health data ( ICD-10 and ICD-9 codes, outcomes, date and type of death, etc.). A few of these information systems, which are linked to each other presented below:

- *Population registry –* The population register is the information system designed to create a single centralized information database on the actual number of individuals assigned to each health organization providing healthcare, as well as for registration of birth and death certificates. Registered patients have their unique population registry number (**RPN**), which is not known by patients or any other health workers. This RPN ID is also not used for any other common reason. During the first patients visits to the primary care physicians and get registered, the system will generate a unique registry ID. The Republican Center of Electronic Healthcare (the holder of all electronic data) can provide this registry ID and all other linkage between the systems can be made by this RPN ID.

- *Electronic registry of dispensary patients* - includes several subunits such as registry of chronic kidney disease, national register of diabetes, national registry of tuberculosis patients, electronic register of cancer patients, register of patients with viral hepatitis, and many others, aimed to register all patients undergoing ambulatory treatment with corresponding ICD-10 codes.

- *Electronic Registry of Inpatients -* designed to collect data of treated inpatients and to create a single, centralized, information database of inpatients, containing all the information necessary to finance medical services of the hospitalized inpatients. The system provides access to current information in order to obtain operational, statistical and analytical reporting on inpatient medical care, information on the admission and discharge, provided medical procedures based on ICD-9 codes and provided diagnosis by ICD-10 codes.

The UNHS has been established in 2014, and before functioning, all previous temporary electronic systems and old registries were transferred and/or integrated to new UNEHS.

Detailed information about other health information systems and subsystems can be accessed from the RCEH website (<https://www.ezdrav.kz>).

***Data extraction:*** The RCEH had extracted data from different Information systems per our request. Initially we will evaluate the IS, and define all variables that can be extracted to RAW data. Corresponding to RPN number, date variables (date of birth and date of death if any) from the Population Registry extracted all together. The following variables were extracted from the *Registry of chronic kidney failure*:

| - patient RPN number; - date of birth (age); - gender; - ethnicity; - educational level; - address; - main diagnosis of ICD-10; - concomitant diseases ICD-10 if any; - date of first registered dialysis procedure; - date of the last dialysis; - organization performing the dialysis procedure; | - type of dialysis (PD or HD); - date of kidney transplant surgery; - type of donation (live or cadaveric); - date of death; - main cause of death; |
| --- | --- |

The date of the first dialysis procedure and the date of kidney transplant surgery might be registered to the UNHS retrospectively from previous temporary electronic systems and old registries if they were conducted before 2014. The earliest dialysis was performed on 1st of Feb 1997.

The data may include duplicate observations (duplicate RPN numbers). The reason for duplicates is different ICD-10 codes indicating the same diagnosis, e.g. one patient might has N18 and N18.1 made from different information systems (from Population registry, from Registry of chronic kidney disease or from registry of inpatients). Such duplicate cases removed after the detailed explore of the cases.

**Supplement Table 1**. **Average** **population of Kazakhstan and by region between 2014 - 2018 years**

| Years | 2014 | 2015 | 2016 | 2017 | 2018 |
| --- | --- | --- | --- | --- | --- |
| Akmola oblast | 736,086 | 740,490 | 739,394 | 736,655 | 738,765 |
| Aktobe oblast | 815,771 | 828,682 | 840,244 | 851,695 | 863,674 |
| Almaty oblast | 1,953,230 | 1,934,720 | 1,965,508 | 2,000,372 | 2,028,106 |
| Atyrau oblast | 574,610 | 587,950 | 601,020 | 614,106 | 627,237 |
| West Kazakhstan oblast | 627,070 | 633,518 | 639,246 | 644,220 | 649,626 |
| Zhambyl oblast | 1,091,429 | 1,104,619 | 1,113,028 | 1,116,262 | 1,121,329 |
| Karagandy oblast | 1,373,890 | 1,381,466 | 1,383,772 | 1,381,635 | 1,379,535 |
| Kostanay oblast | 881,268 | 882,688 | 881,470 | 877,375 | 874,205 |
| Kyzylorda oblast | 746,364 | 759,030 | 769,100 | 778,150 | 788,746 |
| Mangystau oblast | 597,137 | 616,808 | 634,799 | 651,571 | 669,258 |
| Pavlodar oblast | 754,354 | 757,194 | 757,804 | 755,934 | 754,354 |
| North Kazakhstan oblast | 573,887 | 570,712 | 566,447 | 560,942 | 556,550 |
| Turkestan oblast | 2,760,480 | 2,814,388 | 2,859,754 | 1,971,682 | 1,980,499 |
| East Kazakhstan oblast | 1,394,927 | 1,395,742 | 1,392,794 | 1,386,657 | 1,381,136 |
| Nur-Sultan city | 833,611 | 862,694 | 922,638 | 1,001,634 | 1,054,480 |
| Almaty city | 1,574,171 | 1,672,105 | 1,727,037 | 1,776,651 | 1,828,325 |
| Shymkent city | N/A | N/A | N/A | 932,234 | 980,627 |
| Total | **17,288,285** | **17,542,806** | **17,794,055** | **18,037,776** | **18,276,499** |

**Supplement Figure 1.** Prevalence, incidence and mortality of dialysis patients by rural and urban regions for 2014-2018 years

**
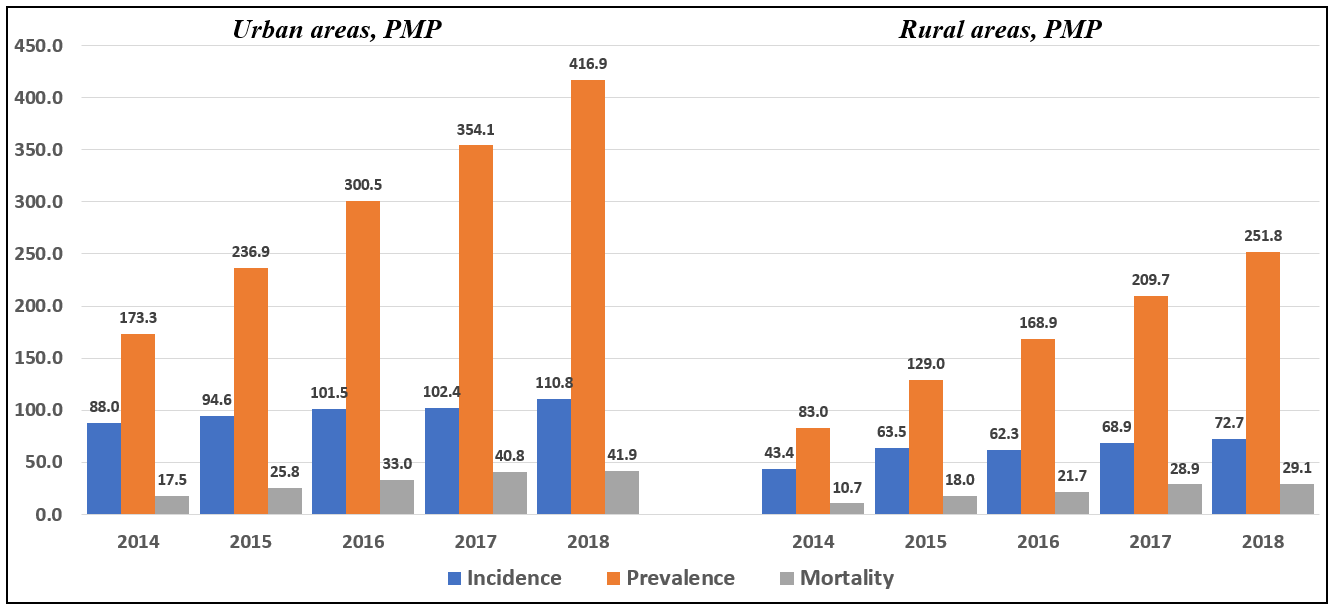
**

**Supplement Figure 2**. The number of new cases by age category for the period 2014-2018.


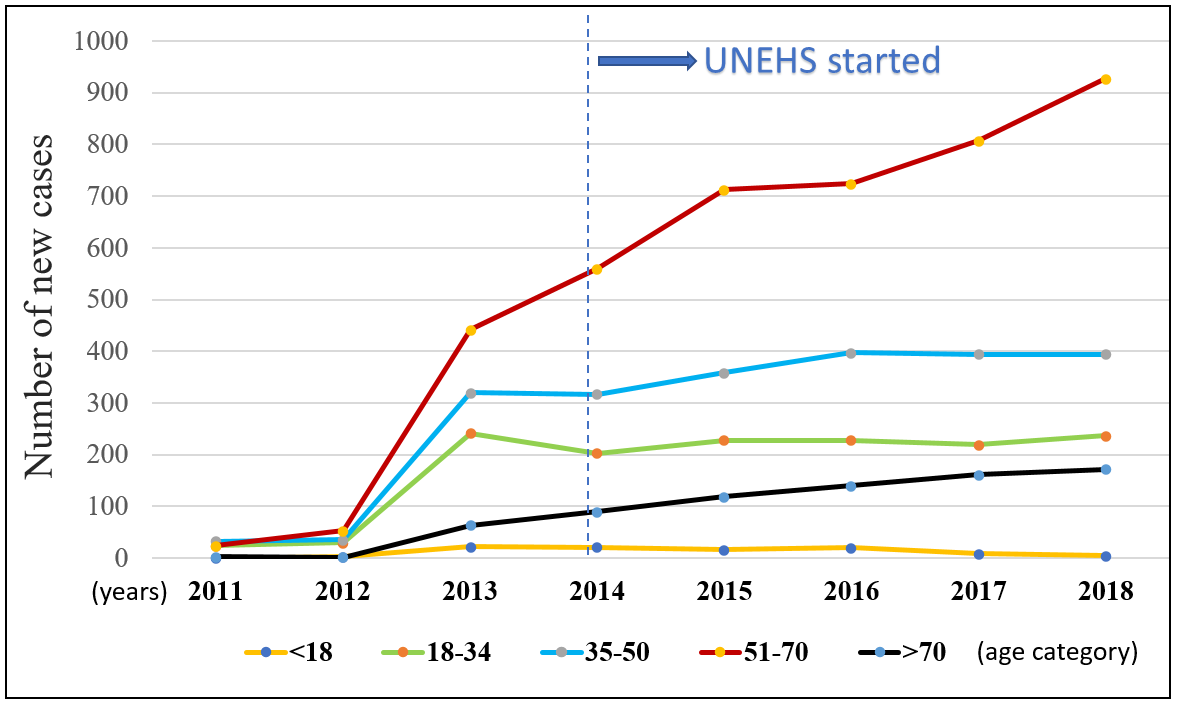


**Supplement Figure 3**. Prevalence (A), Incidence (B) and Mortality (C) of Dialysis patients by Regions in 2017


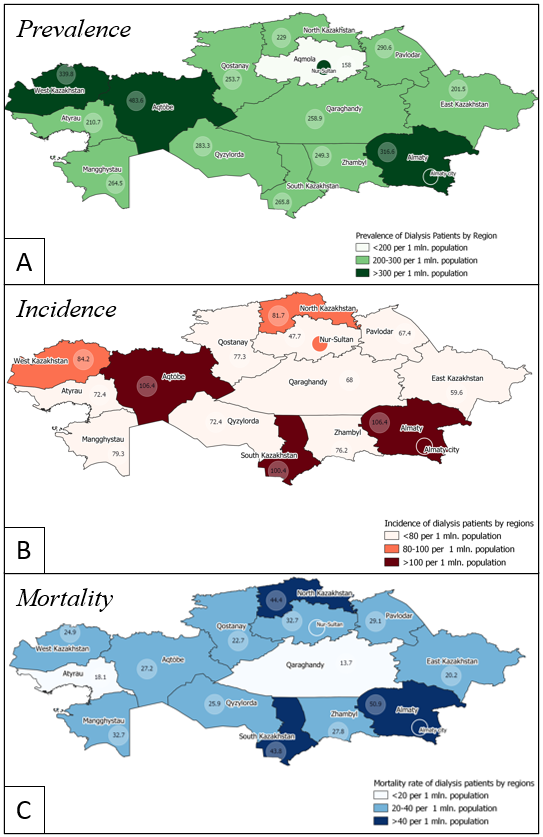


**Supplement Figure 4**. Adjusted survival probability on dialysis by gender (panel A, adjusted for age) and ethnicity (panel B, adjusted for age and gender).


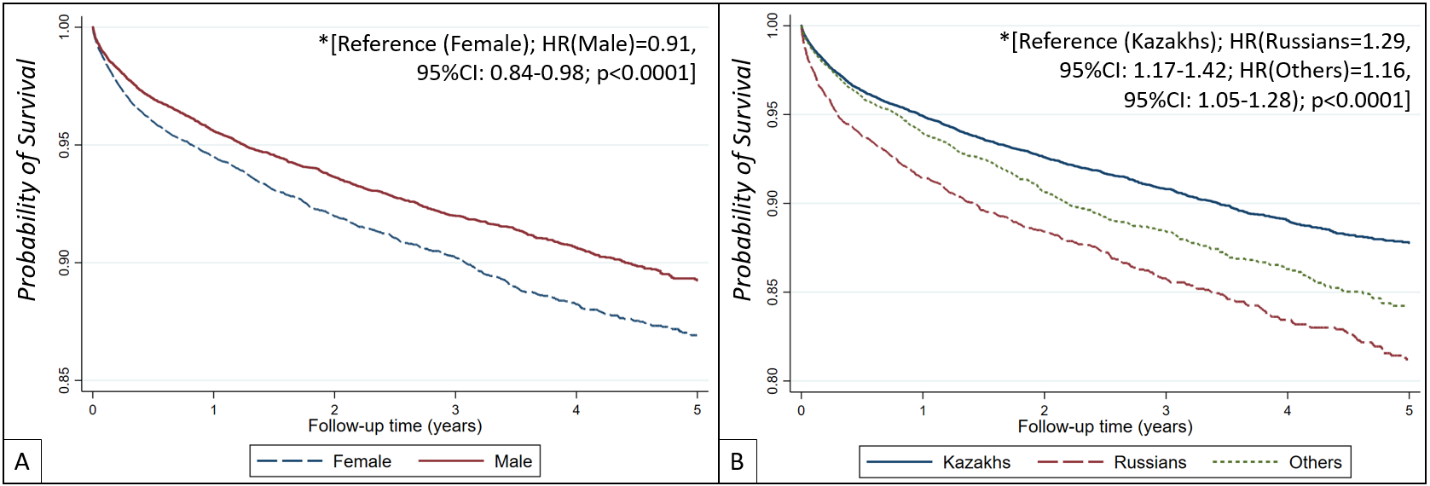


*Adjusted Cox proportional hazard regression analysis.

Abbreviations: HR – hazard ratio.

**Supplement Figure 5. Adjusted** cumulative incidence of transplant censored all-cause death on dialysis by gender (panel A, adjusted for age) and ethnicity (panel B, adjusted for age and gender).

**
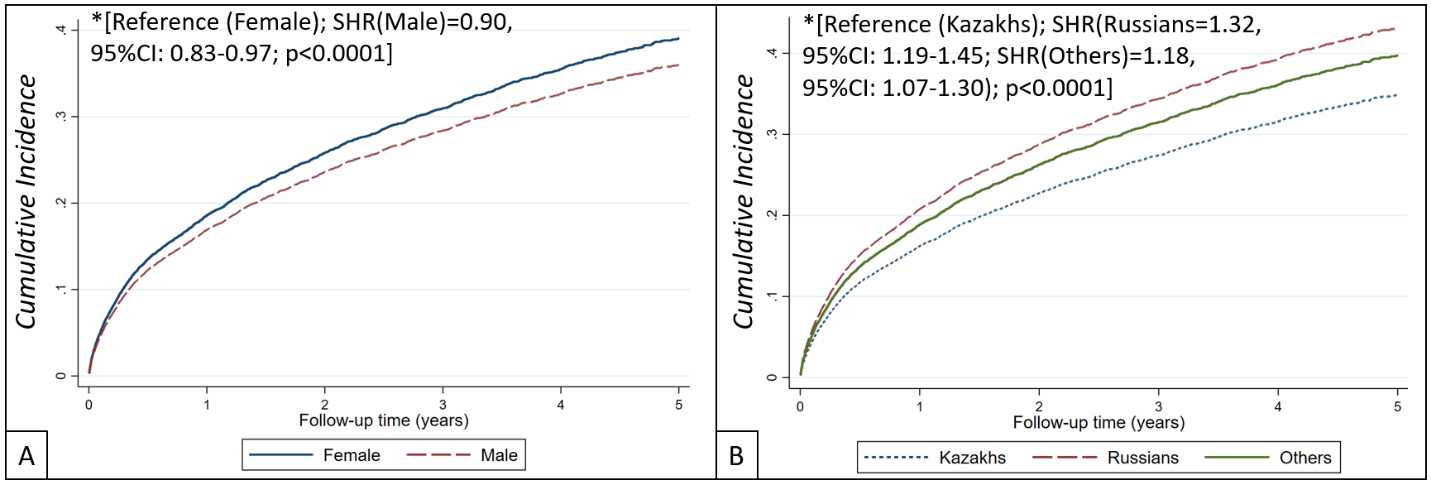
**

*Adjusted competing risk regression analysis.

Abbreviations: SHR – subdistribution hazard ratio.

**Supplement Figure 6.** Cumulative incidence of transplant censored all-cause death on dialysis by age category (A) and education level (B).


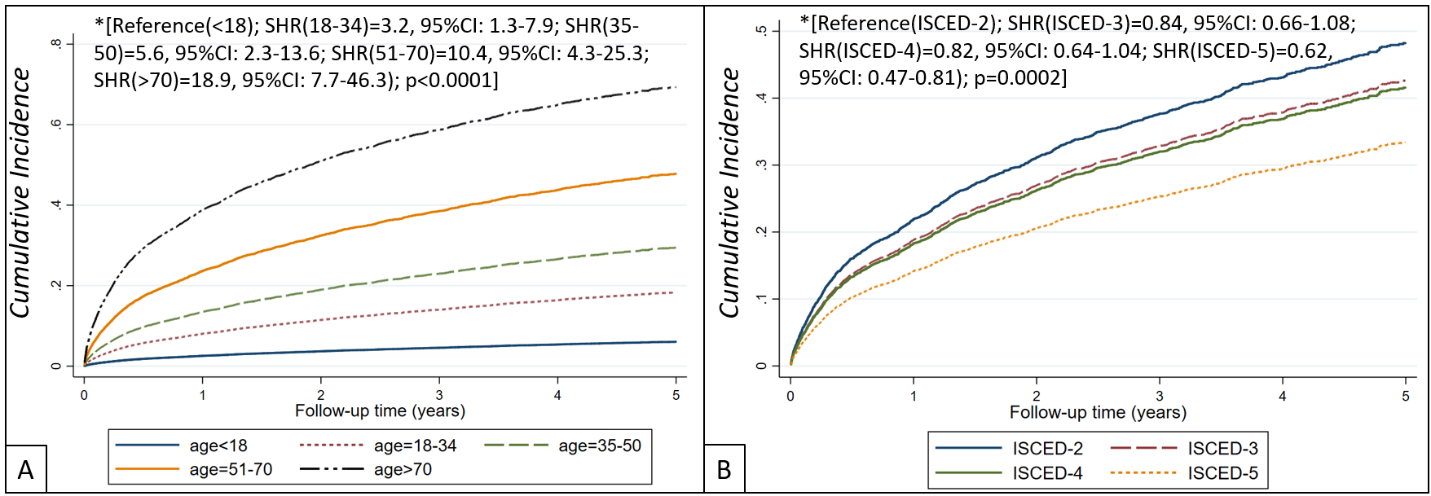


*Unadjusted competing risk regression analysis.

Abbreviations: ISCED - International Standard Classification of Education; SHR – subdistribution hazard ratio.
